# Transmission Pattern of Drug-Resistant Tuberculosis and Its Implication for Tuberculosis Control in Eastern Rural China

**DOI:** 10.1371/journal.pone.0019548

**Published:** 2011-05-12

**Authors:** Yi Hu, Barun Mathema, Weili Jiang, Barry Kreiswirth, Weibing Wang, Biao Xu

**Affiliations:** 1 Key Laboratory of Public Health Safety, Ministry of Education, and Department of Epidemiology, School of Public Health, Fudan University, Shanghai, China; 2 Tuberculosis Center, Public Health Research Institute, Newark, New Jersey, United States of America; St. Petersburg Pasteur Institute, Russian Federation

## Abstract

**Objective:**

Transmission patterns of drug-resistant *Mycobacterium tuberculosis* (MTB) may be influenced by differences in socio-demographics, local tuberculosis (TB) endemicity and efficaciousness of TB control programs. This study aimed to investigate the impact of DOTS on the transmission of drug-resistant TB in eastern rural China.

**Methods:**

We conducted a cross-sectional study of all patients diagnosed with drug-resistant TB over a one-year period in two rural Chinese counties with varying lengths of DOTS implementation. Counties included Deqing, with over 11 years' DOTS implementation and Guanyun, where DOTS was introduced 1 year prior to start of this study. We combined demographic, clinical and epidemiologic information with IS*6110*-based restricted fragment length polymorphism (RFLP) and Spoligotyping analysis of MTB isolates. In addition, we conducted DNA sequencing of resistance determining regions to first-line anti-tuberculosis agents.

**Results:**

Of the 223 drug-resistant isolates, 73(32.7%) isolates were identified with clustered IS*6110*RFLP patterns. The clustering proportion among total drug-resistant TB was higher in Guanyun than Deqing (26/101.vs.47/122; *p*,0.04), but not significantly different among the 53 multidrug-resistant isolates (10/18.vs.24/35; *p*,0.35). Patients with cavitary had increased risk of clustering in both counties. In Guanyun, patients with positive smear test or previous treatment history had a higher clustering proportion. Beijing genotype and isolates resistant to isoniazid and/or rifampicin were more likely to be clustered. Of the 73 patients with clustered drug-resistant isolates, 71.2% lived in the same or neighboring villages. Epidemiological link (household and social contact) was confirmed in 12.3% of the clustered isolates.

**Conclusion:**

Transmission of drug-resistant TB in eastern rural China is characterized by small clusters and limited geographic spread. Our observations highlight the need for supplementing DOTS with additional strategies, including active case finding at the village level, effective treatment for patients with cavities and drug susceptibility testing for patients at increased risk for drug-resistance.

## Introduction

Drug-resistant *Mycobacterium tuberculosis* (MTB) once confined to hospitals and institutional settings,is now widespread in some communities and stands to undermine global tuberculosis control efforts. Of particular concern is the occurrence of multidrug-resistant tuberculosis (MDR-TB), defined as resistance to at least isoniazid and rifampicin. MDR-TB patients respond poorly to conventional first-line therapy, are costly to manage, and typically remain infectious for prolonged periods of time due to inefficient bacillary clearance [1], [2], [3]. Clearly, a better understanding of drug-resistant TB epidemiology is paramount to inform evidence-based control strategies for MDR-TB.

Drug-resistance is associated with a number of factors including poor adherence to anti-TB treatment[4]. MDR-TB comes about as a result of the stepwise accumulation mutations in drug-resistance conferring genes. Previously, drug-resistant MTB strains were thought to be less infectious and less likely to cause disease when compared to their drug-susceptible counterparts [5]. However, recent studies have shown that they are able to transmit and cause disease as often as drug-susceptible organisms [6], [7], [8]. In addition to de novo acquisition, primary transmission of already resistant organisms may be fueling the ongoing MDR-TB epidemic [9], [10].

Directly observed treatment, short course (DOTS) is a cost-effective strategy to control TB, where standardized chemotherapy observed by trained health providers is the key element for treatment compliance and in preventing drug-resistance. However, MDR-TB cases are increasingly reported in DOTS-covered areas [10], . In China, DOTS-based TB control programs have been implemented comprehensively since its introduction over 15 years ago and by 2007 DOTS coverage in China had reached almost 100% [13]. Despite DOTS penetration, there remains significant increase in prevalence of MDR-TB particularly in rural areas. China is considered one of the “hotspot” regions for drug-resistant TB by WHO [14] and accounts for a quarter of the global burden.

A recent study conducted in eastern rural China reported a significantly higher proportion of MDR-TB in regions with long-term DOTS coverage when compared to short-term DOTS covered areas [15]. In addition, drug-resistant MTB circulating in these communities in rural China was strongly associated with specific resistance conferring mutations [16]. Furthermore, a major subgroup within the Beijing family[17] and strains with *katG*315T [18] possess an increased predisposition to develop MDR and transmission in rural China. Based on these observations, we sought to systematically determine the extent of transmission of drug-resistant MTB in these two counties, with varied lengths of DOTS implementation. Furthermore, we examined transmission of drug-resistant TB at the phenotypic and genotypic level, explored risk factors for the recent transmission, as well as assessed relative geographic distances between patients infected with “clustered” MTB IS*6110* RFLP genotypes in two counties, in an attempt to provide the knowledge base to understand the epidemic of drug-resistant TB as well as to inform TB control activities in rural China.

## Materials and Methods

The study was approved by the Institutional Review Board of Fudan School of Public Health. Written informed consent was obtained from all the participants.

### Study sites

Jiangsu Province and Zhejiang Province are located in eastern China and border each other. Two counties Deqing and Guanyun were selected separately from these two provinces as the study sites. The selection of study sites was based on the comparable socioeconomics, demographics, general health systems, capacity and willingness of local partners. While Deqing implemented the DOTS-based National TB Control Program guidelines 11 years ago, Guanyun adopted the DOTS strategy less than 1 year prior to start of this study. In both counties, the county TB dispensary is the only designated health facility for TB diagnosis, treatment and case management. Due to limited resources, bacterial culturing and drug susceptibility testing (DST) were not routinely performed. TB treatment was based on the standardized therapy using 1^st^-line anti-TB agents.

### Bacterial isolates and clinical data

In this population-based epidemiological study, a total of 399 diagnosed pulmonary TB patients, 182 in Deqing and 217 in Guanyun, registered at local TB dispensaries during 12 months consecutive in 2004–2005 were enrolled. All patient specimens at TB dispensaries were submitted to the microbiology laboratory in School of Public Health, Fudan University for culturing and identification. After identification by implanting colonies separately in PNB and TCH containing culture media, 387 isolates was defined as MTB by presenting TCH positive and PNB negative. MTB isolates were further examined for their susceptibilities to isoniazid (INH), streptomycin (STR), ethambutol (EMB) and rifampicin (RIF) by proportion method [19]. Results from culturing and drug susceptibility testing as well as demographic and clinic information were available for 164 (90.1%) isolates in Deqing and 187 (86.2%) isolates in Guanyun, respectively. These MTB isolates were included in the present study.

### Genotyping

Genotyping was performed on each isolate using both IS*6110*-based Restricted Fragment Length Polymorphism (RFLP) and Spoligotyping. IS*6110* RFLP was performed according to methods described by van Embden and coworkers [20]. Briefly, chromosomal DNA was digested with restriction endonuclease *Pvu*II and hybridized with a probe prepared from a 245-bp PCR product of IS*6110*. IS*6110* Southern blot hybridization pattern was visualized using a commercial kit (Maarssen, The Netherlands). The DNA fingerprint patterns of the isolates were compared both by Gelcompar software (version 3.lb, Applied Maths, Kortrijk, Belgium) and by visual examination. A molecular “cluster” was defined as two or more persons whose organisms had identical IS*6110* RFLP patterns, while the organisms with orphan IS*6110* RFLP pattern was defined as “unique” strain. Typically, clustered strains indicate recent transmission while unique strains indicate reactive disease from a remote infection.

Spoligotyping was performed to differentiate the Beijing family from other genotypes. The spacer between the direct repeats in the target region was amplified using two 18 bp nucleotide primers as described elsewhere [21]. The PCR products were hybridized to commercial membrane (Isogen Bioscience BV, Maarssen, The Netherlands). Hybridized DNA was detected using an enhanced chemiluminescene kit from Isogen Bioscience BV (Maarssen, The Netherlands), which resulted in patterns for each strain reminiscent of a ‘barcode’. Strain family was determined by comparing Spoligotyping patterns with the SpoligDB4 database [21]. Beijing family MTB was defined as those hybridizing the last nine spacer oligonucleotides (spacers 35 to 43) of the Spoligotyping pattern.

### DNA sequencing

We targeted the 5′ and 3′ flanking resistance determining regions of *rpoB*(Genebank assession No.L27989), *katG*(Genebank assession No.X68081), *rpsL*(Genebank assession No.L08011) and *embB*(Genebank assession No.U68480), using oligonucleotide primers previous described[16]. Targeted PCR products were sequenced using an Applied Biosystem 3730/3730×l DNA analyzer. DNA sequencing data was aligned manually using Sequencer 4.7 software (Gene Codes Corporation). All the nucleotide data has been submitted and deposited in GenBank.

### Statistical analysis

Descriptive analyses were performed using SPSS 11.0 (SPSS, Chicago, IL, USA). ANOVA and 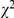
tests were used to examine difference in baseline information between the two rural counties. Univariate and multivariate analysis were conducted using binary logistic regression model. Odds ratio (OR) and 95% confidence interval (CI) were reported after adjusting for age, sex and counties. Statistical significance was defined as *p* value of 0.05 or less.

## Results

Detailed characteristics of studied patients and the drug-resistant pattern and the genetic mutation of MTB isolates have been reported previously [15], [16]. Summary characteristics of subjects were shown in Table 1. No statistically significant differences were observed in the socio-demographic and clinical features of patients from the two counties.

**Table 1:** Demographic and clinic characteristics of patients and drug-resistant patterns at diagnosis in Deqing and Guanyun.

Variables	Deqing (*n* = 164)		Guanyun (*n* = 187)		χ^2^	*p*
	No.	%	No.	%		
Male (sex)	109	66.5	128	68.4	0.157	0.69
Age (mean±SD)	48.44±18.96		48.20±19.76		0.045^a^	0.83
Cavity	22	13.4	33	17.6	1.184	0.28
Smear positive	110	67.1	129	69.0	0.147	0.70
BMI value (mean±SD)	18.33±2.62		18.90±3.00		3.579^a^	0.06
Previously treated	42	25.6	51	27.3	0.124	0.73
Drug-resistant pattern at diagnosis						
Pan-drug sensitive	63	38.4	65	34.8	4.089	0.13
MDR-TB	18	11.0	35	18.7		
Other drug-resistance	83	50.6	87	46.5		

^a^F value for ANOVA test.

**Figure 1:**
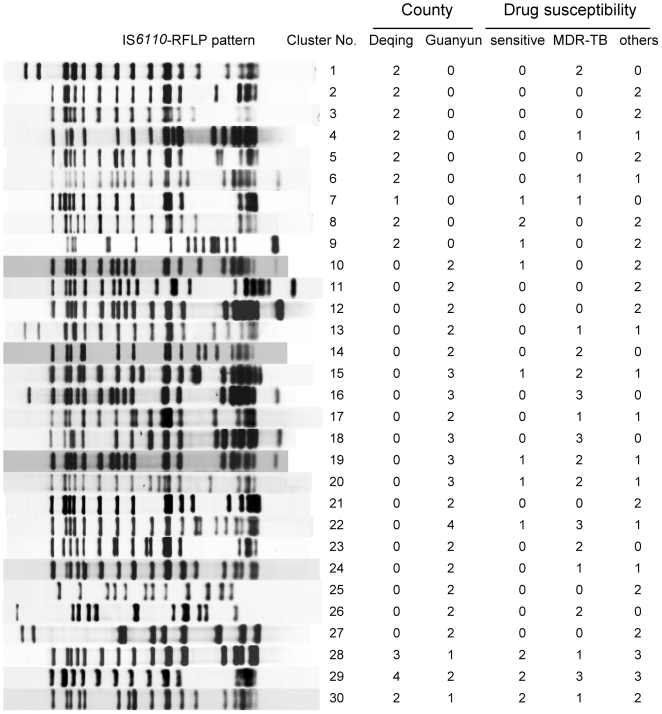
The clustering patterns of IS*6110* RFLP and their distribution in counties and 1st-line drug susceptibilities groups. The isolates in the counties represented the clustered drug-resistant MTB with the identical IS*6110* RFLP patterns.

Of the 223 drug-resistant MTB isolates, a total of 180 IS*6110* RFLP genotype patterns were observed, with 30 clusters composed of 73 drug-resistant isolates (32.7%). Of these clusters, 19 clusters were composed solely by 40 drug-resistant isolates, and the other 11 clusters were shared with 33 drug-resistant and 15 pan-drug sensitive isolates. The remaining 150 drug-resistant isolates (67.3%) gave the unique fingerprint patterns. A total of 12 and 21 clusters were observed in Deqing and Guanyun, with three IS*6110* RFLP patterns shared between the two counties. The clustering proportion of drug-resistant TB was significantly higher in Guanyun than that in Deqing (26/101.vs.47/122; *p*, 0.04), but there was no significant difference for MDR-TB (10/18.vs.24/35; *p*, 0.35) (Figure 1). Results from Spoligotyping showed that 163 (73.1%) isolates belonged to Beijing family. Spoligotype-based lineages for 46 of the remaining 60 isolates were as follows: Family 33 (17/223 or 7.6%), T lineage (15/223 or 6.7%), Haarlem (5/223 or 2.2%), LAM (8/223 or 3.6%) and EAI (1/223 or 0.4%). Twelve isolates shared identical Spoligotyping patterns, of which 2 were in family 33 shared Spoligotyping International Type (SIT) 54 (Octal value 7777 7777 7763 771), 2 and 4 isolates in T1 shared SIT 52(Octal 7777 7777 7760 731) and SIT53 (Octal value 7777 7777 7760 771), 2 and 2 in LAM family shared SIT 1755 (Octal 6777 7760 7560 771) and new SIT(Octal value 7777 7760 3560 731) (Table S1).

Results from the multivariate analysis are shown in Table 2. Stratified by county, drug-resistant isolates within Beijing family had a significantly higher clustering proportion than those from the other strain families (Deqing: OR, 6.18; 95%CI, 1.332–28.64; Guanyun: OR, 3.75; 95%CI, 1.396–10.06). Drug-resistant isolates from the patients with cavitary disease were more likely to be clustered than those without cavitation (Deqing: OR, 4.50; 95%CI, 1.421–14.26; Guanyun: OR, 2.85; 95%CI:1.195 –6.782). In Guanyun, previous treatment history (OR, 5.66; 95%CI, 2.332–13.73) and positive sputum smear status (OR, 2.98; 95%CI, 1.191–7.441) were independently associated with clustering among drug-resistant MTB isolates (Table 2).

**Table 2:** Univariate and multivariate analysis on the factors influencing the clustering proportion of drug-resistant TB (*n* = 223).

Variable	Deqing county						Guanyun county					
	Total	Clustered	cOR	aOR^a^	*p* a	95%CI^a^	Total	Clustered	cOR	aOR^a^	*p* a	95%CI^a^
BMI												
>18.5	51	10 (19.6)	1.00	1.00			58	19(32.8)	1.00	1.00		
≤18.5	50	16 (32.0)	1.93	1.92	0.17	0.760–4.820	64	28(43.8)	1.60	1.77	0.15	0.818–3.840
TB contact												
No	88	22 (25.0)	1.00	1.00			92	34(37.0)	1.00	1.00		
Yes	13	4 (30.8)	1.33	1.38	0.63	0.382–4.963	30	13(43.3)	1.30	1.37	0.48	0.579–3.235
Sputum smear test												
negative	28	6 (21.4)	1.00	1.00			38	8 (21.1)	1.00	1.00	0.02	
positive	73	20(27.4)	1.38	1.38	0.54	0.488–3.910	84	39(46.4)	3.25	2.98	0.01^b^	1.191–7.441
Cavity												
No	86	18(20.9)	1.00	1.00			92	30(32.6)	1.00	1.00		
Yes	15	8 (53.3)	4.32	4.50	0.01^b^	1.421–14.26	30	17(56.7)	2.70	2.85	0.02 b	1.195–6.782
Treatment history												
New	68	17(25.0)	1.00	1.00			79	21(26.6)	1.00	1.00		
Prior treated	33	9 (27.3)	1.13	1.14	0.78	0.443–2.945	43	26(60.5)	4.22	5.66	0.01 b	2.332–13.73
Strain family												
Non-Beijing	27	2 (7.4)	1.00	1.00			33	6(18.2)	1.00	1.00		
Beijing genotype	74	24(32.4)	6.00	6.18	0.02^b^	1.332–28.64	89	41(46.1)	3.84	3.75	0.01^b^	1.396–10.06

a OR and 95% CI was adjusted for age and sex in the binary logistics regression model.

b *p*<0.05.

Clustering proportion among drug-resistant MTB isolates was compared to the 128 drug-susceptible MTB isolates collected simultaneously. Isolates resistant to INH (48.1%.vs. 30.5%; *p*, 0.008; OR, 2.01; 95%CI, 1.204–3.369), RIF (53.8%.vs.30.5%; *p*, 0.003; 2.62; 95%CI, 1.382–4.980) and MDR-TB (64.2%.vs. 30.5%; *p*, 0.0001; OR, 3.87; 95%CI, 1.924–7.764) were more likely to be clustered compared to drug-susceptible isolates (Table 3). The association between drug-resistance conferring genotype and clustering was investigated by comparing the clustering proportion between drug-resistant genotype and wide type isolates. Among INH resistant isolates, *katG* 315Thr alleles (73.0%.vs18.0%; *p*, 0.0001; OR, 12.7; 95%CI: 6.357–14.80) were strongly associated with clustering. No other alleles conferring resistance to RIF, STR and EMB were associated with clustering.

**Table 3:** Clustering proportion between the MTB isolates with different drug-resistant pattern and genetic mutation (*n* = 223).

		No.of isolates		crude	Adjusted OR^b^	*p* b	95%CI^b^
		Total	Clustered(%)	OR^a^			
**Isolates resistant to:**							
Sensitive		128	39(30.5)	1.00	1.00		
INH		131	63(48.1)	2.05	2.01	0.008^c^	1.204–3.369
RIF		65	35(53.8)	2.66	2.62	0.003^c^	1.382–4.980
STR		115	26(22.6)	0.67	0.68	0.19	0.376–1.215
EMB		42	12(28.6)	0.91	0.90	0.79	0.405–1.988
MDR-TB		53	34(64.2)	4.08	3.87	0.0001^c^	1.924–7.764
Other combinations		170	39(22.9)	0.82	0.82	0.15	0.634–1.070
**Drug-resistant isolates with mutation in:**							
*katG*							
* wt*		50	9 (18.0)	1.00	1.00		
* others*		7	0 (0)	-	-	-	-
* 315Thr*		74	54(73.0)	12.3	12.7	0.0001^c^	6.357–14.80
*rpoB*							
* wt*		5	1(20.0)	1.00	1.00		
* others*		23	10 (43.5)	4.22	4.88	0.12	0.597–19.298
* 531Leu*		37	24 (64.9)	8.71	8.50	0.08	0.771–93.65
*rpsL*							
* wt*		37	9 (24.3)	1.00	1.00		
* others*		18	2 (11.1)	0.33	0.55	0.49	0.099–3.035
* 43Arg*		60	15(25.0)	1.04	1.41	0.51	0.511–3.865
*embB*							
* wt*		21	5(23.8)	1.00	1.00		
* 306lle*		8	2(25.0)	1.27	1.19	0.87	0.164–8.584
* 306Val*		13	5(38.5)	1.80	1.59	0.14	0.673–9.100

Note: INH,isoniazid; RIF,rifampicin; STR, streptomycin; EMB, ethambutol.

a cOR: crude odds ratio were calculated by comparing the clustering proportion between drug- resistant MTB isolate and drug-susceptible isolate in binary logistic regression model.

b aOR: adjusted odds ratio and 95%CI were calculated by comparing the clustering proportion between drug-resistant TB isolate and drug-susceptible isolate, adjusted by age, sex, and counties of the subjects in a binary logistic regression model.

c *p*<0.05.

**Figure 2:**
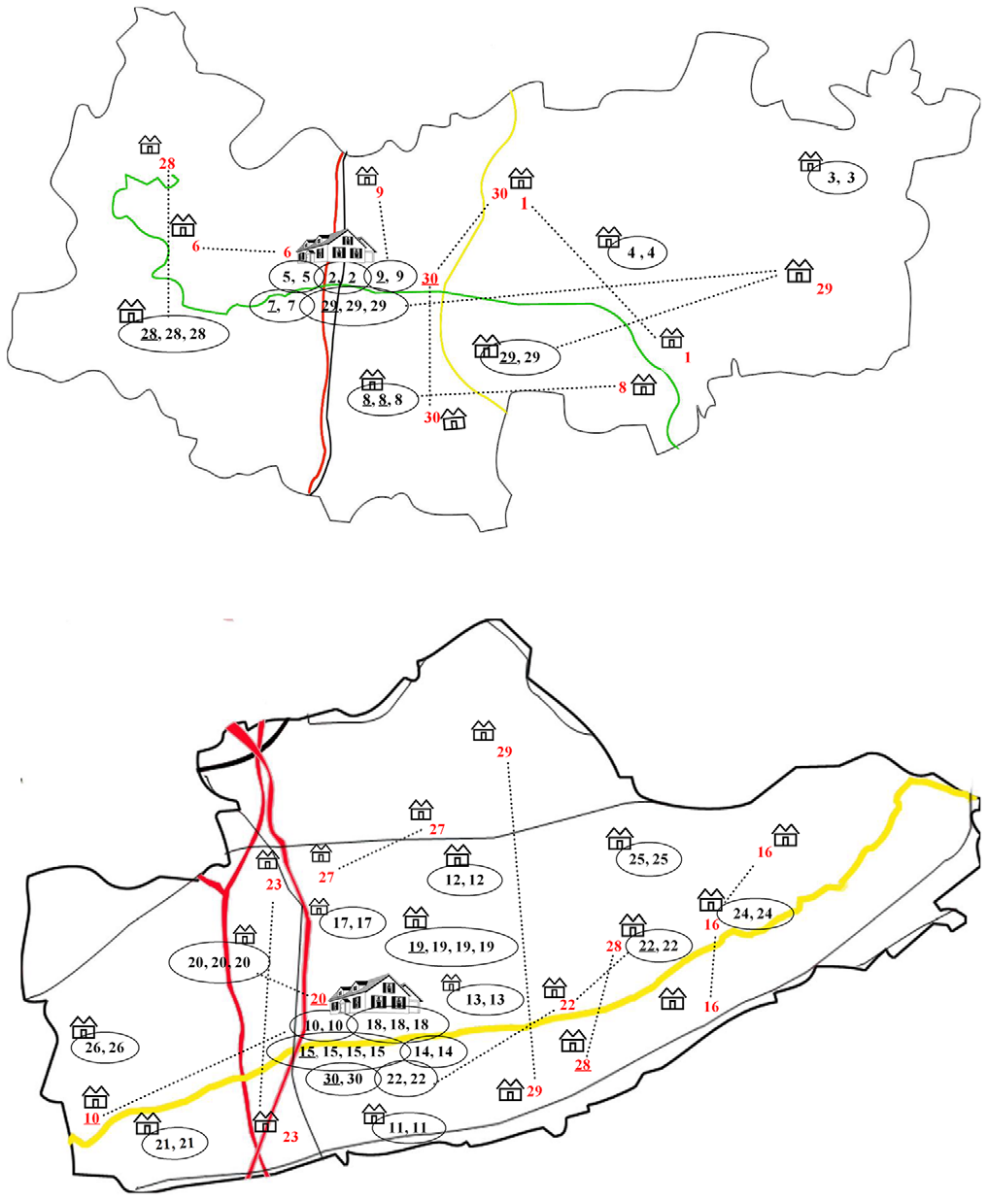
The geographic distribution of the patients with the clustered MTB isolates in Deqing(upper) and Guanyun(lower). Note: cottage represented the township; vile represented the central area of the counties; No. marked the IS*6110* RFLP clusters; the underlined No. represented the drug-susceptible MTB isolates; Eclipse marked the clustered isolates distributed in the same township; the dotted line connected the clustered isolate without geographic correlation.

Epidemiological links within drug-resistant clusters and geographic distribution based on patients' residence are depicted in Figure 2. Of the 30 clusters (i.e. identical IS*6110* RFLP patterns), 9 in Deqing and 16 in Guanyun were geographically restricted, originating from the same or neighboring village in the same town. The town central of Deqing and Guanyun contained 5 and 6 clusters respectively, involving 8 and 13 drug-resistant patient isolates. The isolates from the 3 clusters in Deqing and 5 clusters in Guanyun were obtained from different localities. From potential contact perspective, 4 of 26 (15.4%) clustered isolates from Deqing and 5 of 47 (10.6%) clustered isolates from Guanyun were obtained from family members (household contact), friends or working colleagues (social contact). No epidemiological links or geographic correlation was established for 9 clustered drug-resistant isolates (34.6%) in Deqing and 11 clustered drug-resistant isolates (23.4%) in Guanyun.

## Discussion

Transmission patterns of drug-resistant MTB has been well documented in low TB incidence countries, however there is paucity of reports from high TB burden countries like China; this information could be very important in decision-making for TB control. To that end, the present study aimed to describe the transmission of drug-resistant TB in two comparable rural Chinese communities with different duration of DOTS implementation, and to identify risk factors for developing appropriate strategies for prevention and control of drug-resistant TB in eastern rural China.

IS*6110* DNA fingerprinting indicated that the degree of MTB genotypic heterogeneity did not vary significantly between Deqing and Guanyun, with 87 and 86 IS*6110* RFLP patterns respectively. This may be expected given the overwhelming dominance of the Beijing family strain in most regions in China [17], [22]. In addition to its overall prevalence, Beijing family members are increasingly found associated with MDR-TB [23], [24] and enhanced transmissibility in some areas [25], [26]. Similarly in the present study, the majority (73.1%) of drug-resistant TB were from members of the Beijing family. Drug-resistant TB isolates within Beijing family showed a relatively high level of clustering (40.0%), suggesting active transmission. Furthermore, three Beijing IS*6110* RFLP clusters were simultaneously observed in Deqing from Zhejiang Province and Guanyun from Jiangsu Province, possibly due to its inter-provincial transmission. This tendency may suggest high transmissibility potential for some specific members of Beijing family [17]. The active transmission of such subgroups may contribute to the ongoing epidemic of drug-resistant TB in rural China and highlights the need for better control measures to prevent the primary transmission.

In Guanyun, a county with recent DOTS implementation, most clusters were among cases with previous treatment history. In addition, cavitary lesions and sputum smear positivity (indicating infectiousness) were significantly more common among the drug-resistant cases with clustered genotypes than those infected with unique genotypes. Our findings are consistent with those reported by others [27], [28]. Typically, patients with cavitary disease harbor higher bacillary burdens, thereby increasing the likelihood of selecting mutants during therapy [29], [30]. Smear positivity would similarly indicate higher bacillary burden and increased infectiousness. The high proportion of clustering pattern in previously treated cases is also troubling as it indicates that inadequate previous treatment increasing the risk of drug-resistance and primary transmission. While in Deqing where DOTS had been implemented for over 15 years, only patients with cavities had increased risk for clustering. Previous treatment history wasn't found a risk factor for clustering as it was in Guanyun County. The differences between the counties might indicate population-level effects of long-term regulated and standardized TB treatment (i.e. DOTS). However, we did not observe the significant difference in the clustering proportion among MDR-TB isolates between two counties, suggesting that without routine bacteriologic-based diagnosis and drug susceptibility testing and without regulated treatment for drug-resistant TB, DOTS itself might not be able to prevent the risk of community-level MDR-TB transmission. Therefore, MDR-TB control programs in China may be modified according to local settings and needs additional strategies such as establishing referral system for patients at high risk of drug-resistant TB, improving ability of diagnosis for drug-resistance in TB clinics and hospitals, and providing individualized treatment for drug-resistant TB patients.

Resistance to anti-TB drugs can develop as a result of treatment failure (acquired resistance), but it can also occur as a result of infection with drug-resistant strains (primary resistance). Among drug-resistant TB patients in our study, INH resistant, RIF resistant and MDR-TB had a significantly higher clustering proportion compared to the drug-susceptible MTB isolates, highlighting the significance of the primary resistance. Comparably, the reactivation from the remote infection might be the main reason for the STR and EMB resistance. A high clustering proportion of *katG*315Thr allele was observed in this study. This allele was associated with MDR-TB as reported elsewhere [18], [31], [32]. Therefore, this allele may be the major genotype responsible for the recent transmission of MDR-TB and INH resistant strain in the studied areas. The different epidemic mechanism for drug-resistant TB implies that tailored strategies are needed for in control of drug-resistant TB. Considering the transmissibility of INH and RIF resistant TB, susceptibility testing of INH and RIF should be provided, and fast diagnosis of drug-resistance is paramount.

The geographic distribution of isolates from clusters containing drug-resistant TB is consistent with the distribution of rural population. Most clones of drug-resistant strains were found to infect small groups of patients. This suggests that active transmission of drug-resistant MTB strain may be limited in Chinese rural communities. Furthermore, a relative high proportion of patients in the clusters lived in the same or neighboring villages. TB patients are usually weak and unable to work, and they have to take treatment for 6–8 months at home. Thus, the scope of their activity is limited, especially in rural China, which might limit the contacts within the same village and makes transmission circle restricted at the village and neighborhood level.

Although clusters came from a wide geographic area, a high level of clustered MTB isolate was observed in the central districts. The central district in rural China commonly has the highest population density and also serves as centers for cultural and social activities. Furthermore, the central districts contain county-level hospitals and TB dispensaries for the diagnosis and treatment of TB. These conditions in central areas may facilitate transmission of MTB. In addition, the present study also noted clustered isolates scattered from different localities, where no clear epidemiological link was identified. Lack of epidemiological links, in the context of clustering, may indicate the existence of wide spread endemic MTB strains. Of note, our one-year study period as well as the passive case detection strategies may have missed intermediate cases in the transmission chain. Our primary genotyping method, IS*6110* RFLP has long served as a gold standard technique with highest discriminatory power for high-copy number isolates including Beijing genotype isolates. However, some recent studies have pointed to clustering misclassification using IS*6110* RFLP alone [33], [34], [35]. Therefore to better define the genotypes of MTB in certain settings, the future studies may benefit from combining IS*6110* RFLP with alternative genotyping methods such as 24-loci variable number tandem repeat analysis.

Adoption of DOTS to prevent the generation of drug-resistant strains and careful introduction of second-line drugs to treat patients with MDR-TB are the top priorities for proper containment of MDR-TB. As reported above in rural China, the transmission pattern of drug-resistant TB was sporadic distribution and in small groups. The spread of drug-resistance may be restricted due to limited social activity of rural populations. Based on our data, anti-TB intervention should focus mainly on individuals proximal to the infectious TB case, rapid identification of drug-resistance, and include active case finding strategies.

## Supporting Information

Table S1 Spoligotyping pattern and drug-resistant pattern and genetic mutation of drug-resistant MTB isolates with non-Beijing family. Note: SIT, Spoligotyping International Type; wt, wide type. ^a^Spotclust program-assigned clade. ^b^Probaiblity that the Spoligotyping pattern belongs to the clades. ^c^Sequence of drugs was Isoniazid, Rifampin, Streptomycin, Ethambutol; R, resistant; S, susceptible.
(DOC)
