# Supplementary material for: Transmission Pattern of Drug-Resistant Tuberculosis and Its Implication for Tuberculosis Control in Eastern Rural China
Source: PLoS One. 2011 May 12;6(5):e19548. doi: 10.1371/journal.pone.0019548 (PMC3093389; doi:10.1371/journal.pone.0019548)
Supplement: Table S1 — Spoligotyping pattern and drug-resistant pattern and genetic mutation of drug-resistant MTB isolates with non-Beijing family. Note: SIT, Spoligotyping International Type; wt, wide type. aSpotclust program-assigned clade. bProbaiblity that the Spoligotyping pattern belongs to the clades. cSequence of drugs was Isoniazid, Rifampin, Streptomycin, Ethambutol; R, resistant; S, susceptible. (DOC) [file pone.0019548.s001.doc]

Table S1 Spoligotyping pattern and drug-resistant pattern and genetic mutation of drug-resistant MTB isolates with non-Beijing family

| Octal Value | Clade ^a^(probability ^b^) | SIT | Drug resistant profile^c^ | Genetic mutation | | | |
| --- | --- | --- | --- | --- | --- | --- | --- |
|  |  |  |  | *katG* | *rpoB* | *rpsL* | *embB* |
| 7777 7777 4413 771 | EAI4 | 456 | SSRS | wt | wt | wt | wt |
| 7777 7777 7146 741 | family33 | New | RRRS | Thr315 | Tyr526 | wt | wt |
| 7777 5737 7763 771 | family33 | New | SSSR | wt | wt | wt | wt |
| 7767 6767 1146 771 | Family33 | New | RSRS | wt | wt | Thr43 | wt |
| 7777 7777 1146 771 | Family33 | New | RSRS | wt | wt | Arg88 | wt |
| 7777 7377 7731 771 | Family33 | New | RSSS | wt | wt | wt | wt |
| 7777 7377 7731 771 | Family33 | New | RSSS | wt | wt | wt | wt |
| 7777 7677 7763 771 | Family33 | 226 | SSRR | wt | wt | Arg43 | Ile306 |
| 7777 7717 7731 771 | Family33 | New | SSRR | wt | wt | Arg43 | Val306 |
| 5133 7777 7763 771 | Family33 | New | SSRS | wt | wt | wt | wt |
| 7777 7737 7731 061 | Family33 | New | SSRS | wt | wt | wt | wt |
| 7777 7777 7763 771 | Family33 | 54 | SSRS | wt | wt | wt | wt |
| 7777 7777 7763 771 | Family33 | 54 | SSSR | wt | wt | wt | wt |
| 5173 7777 7763 771 | Family33 | New | SSSR | wt | wt | wt | wt |
| 7777 3717 7733 571 | Family33 | New | RRRS | wt | Tyr526 | Arg43 | wt |
| 7777 3771 7731 761 | Family33 | New | RSSS | wt | wt | wt | wt |
| 5777 7763 3566 731 | Family33 | New | RSSS | Asn315 | wt | wt | wt |
| 7777 7777 7777 031 | Family33 | New | SSRS | wt | WT | Arg88 | wt |
| 7777 7777 7630 771 | Family33(69.5%),T1(30.5%) | New | RRSS | wt | Arg526 | wt | Ile306 |
| 5577 3316 7710 771 | family33(75%),T1(25.0%) | New | SSSR | wt | wt | wt | wt |
| 5777 3731 6710 771 | Family33(78%),T1(22%) | New | SSRR | wt | wt | wt | wt |
| 7777 7776 0060 771 | Haarlem1 | 164 | SSRS | wt | wt | wt | wt |
| 7777 7777 4000 371 | Haarlem1 | 1275 | SSRS | wt | wt | Arg43 | wt |
| 7777 7777 4020 751 | Haarlem1 | 1629 | SSRS | wt | wt | Arg43 | wt |
| 7777 7777 6000 371 | Haarlem1 | 1498 | RSRS | Thr315 | wt | wt | wt |
| 7777 7777 6000 771 | Haarlem1(97.9%) | New | RSSS | wt | wt | wt | wt |
| 7777 7777 6700 371 | Haarlem3(77.2%),T1(22.7%) | New | SSRR | wt | wt | Thr43 | wt |
| 7757 7737 7700 771 | Haarlem3(77.2%),T1(22.7%) | New | SSSR | wt | wt | wt | wt |
| 7777 7777 7320 771 | Haarlem3(77.2%),T1(22.7%) | 457 | SRRR | wt | Leu531 | Thr43 | wt |
| 5777 7777 7700 771 | Haarlem3(77.2%),T1(22.7%) | New | RSSS | Thr315 | wt | wt | wt |
| 7777 7777 7700 771 | Haarlem3(77.2%),T1(22.7%) | 124 | SSRS | wt | wt | wt | wt |
| 6777 7760 7560 771 | LAM1(50.9%),LAM9(49.1%) | 1755 | SSRS | wt | wt | Arg88 | wt |
| 6777 7760 7560 771 | LAM1(50.9%),LAM9(49.1%) | 1755 | RSSS | Thr315 | wt | wt | wt |
| 6777 7420 7760 771 | LAM1(66.2%),LAM9(33.7%) | 423 | SSRS | wt | wt | Arg43 | wt |
| 7777 7760 7760 771 | LAM9 | 42 | SSRS | wt | wt | wt | wt |
| 7777 7760 3560 731 | LAM9 | New | RRSS | wt | Leu531 | wt | Val306 |
| 7777 7760 3560 731 | LAM9 | New | SSSR | wt | wt | wt | wt |
| 7777 7740 7560 731 | LAM9 | 1355 | SSRS | wt | wt | wt | wt |
| 7777 3740 3760 771 | LAM9 | New | SSRS | wt | wt | Arg43 | wt |
| 7777 7777 7760 771 | T1 | 53 | RRRR | Thr315 | Leu531 | wt | Val306 |
| 7777 7777 7760 771 | T1 | 53 | RRSS | wt | Tyr526 | wt | Ile306 |
| 7777 7777 7760 771 | T1 | 53 | RRSS | Thr315 | Tyr526 | wt | Val306 |
| 7777 7777 7760 771 | T1 | 53 | RSRS | wt | wt | Arg43 | wt |
| 5777 7777 7760 771 | T1 | 334 | RSSR | wt | wt | wt | Ile306 |
| 7777 7777 7760 031 | T1 | 239 | RSRS | wt | wt | Arg43 | wt |
| 7777 7777 7760 731 | T1 | 52 | SRRS | wt | wt | Arg88 | wt |
| 7777 7777 7760 731 | T1 | 52 | SSRS | wt | wt | Arg88 | wt |
| 5777 3777 7760 771 | T1 | New | SSRS | wt | wt | Arg88 | wt |
| 7767 7777 6760 731 | T1 | New | SSRS | wt | wt | wt | wt |
| 7774 7777 7760 731 | T1 | 1056 | SSRS | wt | wt | Arg88 | wt |
| 7776 3777 7760 731 | T1 | New | SSRS | wt | wt | wt | wt |
| 7777 7767 7740 771 | T1 | New | SSSR | wt | wt | wt | wt |
| 5777 7763 3560 731 | T1 | New | SSRS | wt | wt | Arg43 | wt |
| 6777 7777 7760 771 | T1 | 196 | SSRS | wt | wt | Arg43 | wt |
| 7777 7736 7730 771 | T1(78.9%),Family33(21.0%) | New | RRSS | wt | Tyr526 | wt | Ile306 |
| 7577 3737 7730 771 | T1(78.9%),Family33(21.0%) | New | RSSS | wt | wt | wt | wt |
| 7736 3737 7730 771 | T1(78.9%),Family33(21.0%) | New | RSSS | wt | wt | wt | wt |
| 7736 7777 7730 771 | T1(78.9%),Family33(21.0%) | New | RSSS | Thr315 | wt | wt | wt |
| 7777 2737 7730 771 | T1(78.9%),Family33(21.0%) | New | RSSS | Thr315 | wt | wt | wt |
| 7777 7777 7730 771 | T1(78.9%),Family33(21.0%) | New | RSRS | Thr315 | wt | wt | wt |

Note: SIT, Spoligotyping International Type; wt, wide type

^a^ Spotclust program-assigned clade

^b^ Probaiblity that the spoligotyping pattern belongs to the clades

^c^ Sequence of drugs was Isoniazid, Rifampin, Streptomycin, Ethambutol; R, resistant; S, susceptible
